# Persistent Intra-Specific Variation in Genetic and Behavioral Traits in the Raphidophyte, *Heterosigma akashiwo*

**DOI:** 10.3389/fmicb.2015.01277

**Published:** 2015-11-25

**Authors:** Elizabeth L. Harvey, Susanne Menden-Deuer, Tatiana A. Rynearson

**Affiliations:** ^1^Skidaway Institute of Oceanography, University of Georgia, SavannahGA, USA; ^2^Graduate School of Oceanography, University of Rhode Island, NarragansettRI, USA

**Keywords:** phytoplankton, intra-specific variability, behavior, microsatellites, *Heterosigma akashiwo*

## Abstract

Motility is a key trait that phytoplankton utilize to navigate the heterogeneous marine environment. Quantifying both intra- and inter-specific variability in trait distributions is key to utilizing traits to distinguish groups of organisms and assess their ecological function. Because examinations of intra-specific variability are rare, here we measured three-dimensional movement behaviors and distribution patterns of seven genetically distinct strains of the ichthyotoxic raphidophyte, *Heterosigma akashiwo*. Strains were collected from different ocean basins but geographic distance between isolates was a poor predictor of genetic relatedness among strains. Observed behaviors were significantly different among all strains examined, with swimming speed and turning rate ranging from 33–115 μm s^-1^ and 41–110° s^-1^, respectively. Movement behaviors were consistent over at least 12 h, and in one case identical when measured several years apart. Movement behaviors were not associated with a specific cell size, carbon content, genetic relatedness, or geographic distance. These strain-specific behaviors resulted in algal populations that had distinct vertical distributions in the experimental tank. This study demonstrates that the traits of genetic identity and motility can provide resolution to distinguish strains of species, where variations in size or biomass are insufficient characteristics.

## Introduction

The abundance and distribution of phytoplankton populations can have large-scale impacts on primary production and biogeochemical cycling. Understanding the factors that mediate phytoplankton abundance and distribution is difficult, due to the physiological complexity of phytoplankton and the dynamic environment in which they live. Recently, there has been interest in utilizing species functional traits to help resolve the intricacies of plankton ecology, and predict how plankton function in marine food webs may be altered in response to environmental change (Litchman et al., 2007, 2010, 2013; Barton et al., 2013). One of the difficulties in utilizing this method has been in evaluating the importance of a given trait, as well as its plasticity to changing environmental conditions (Litchman and Klausmeier, 2008). Moreover, while this approach has been applied broadly to distinguish among species, less attention has been given to the considerable intra-specific variability known to exist within species (Kremp et al., 2012; Menden-Deuer and Montalbano, 2015; Sassenhagen et al., 2015).

For many phytoplankton species, motility is a key trait that enables phytoplankton to orient, access resources, and avoid predators in a complex ecosystem (Visser and Kiorboe, 2006; Harvey and Menden-Deuer, 2012). Motility is highly variable on an individual level. For example, Menden-Deuer and Grünbaum (2006) found that from a pool of 10,000 cells, swimming speeds of the dinoflagellate *Oxyrrhis marina* ranged from <50 to 800 μm s^-1^. One of the challenges in understanding the impact of motility on phytoplankton population dynamics has been in connecting individuals to resultant population distributions and ultimately population dynamics (Menden-Deuer, 2010). Even small differences in movement behaviors between strains can have significant consequences for the population as a whole. For example, a difference in swimming speed of 20 μm s^-1^ between two strains could result in net displacements of up to two body lengths per second or 1.7 m per day. Thus, even small differences can have considerable ramifications for the abundance and distribution of phytoplankton. Understanding the range in movement behaviors within a single species, and how these behaviors may shift in response to environmental conditions will provide an increased predictive capacity and understanding of phytoplankton population abundance and distributions in the marine environment and the biogeochemical implications.

In addition to considerable variation in behavioral traits, there is extensive genetic variability among phytoplankton as well. Genetic markers such as microsatellites have been used to identify significant genetic variability in field populations of phytoplankton (e.g., Rynearson and Armbrust, 2005; Iglesias-Rodriguez et al., 2006; Nagai et al., 2007; Frommlet and Iglesias-Rodriguez, 2008). In field populations, these high levels of genetic variation could benefit overall population-survival, by maintaining a physiological repertoire to quickly respond to environmental fluctuations through selection on different genotypes, as has been observed recently (Scheinin et al., 2015). Interestingly, clonal diversity has been linked to differences in physiological traits such as genome size (Whittaker et al., 2012), morphology (Saravanan and Godhe, 2010), growth rate (Rynearson and Armbrust, 2004), and toxin composition (Touzet et al., 2007). To date no study has examined whether individual-level genetic variation can be used to identify inherent behavioral diversity in phytoplankton. Such data would provide critical tools to link real-time, *in situ* genetic diversity, with the ecological ramifications of expressed behaviors, such as the distribution of motile species, cell–cell or cell-resource encounter rates, and overall population dynamics.

In order to investigate such an opportunity for linking genetic diversity with behavioral diversity we determined whether differences in motility were reflected in genetic diversity in seven *Heterosigma akashiwo* clones representing a range of geographic origins and age since isolation. *H. akashiwo* (Y. Hada) is a marine raphidophyte that can form ichthyotoxic blooms, causing mortality in both caged and naturally occurring fish populations, and has sublethal effects on invertebrates and marine mammals (Honjo, 1993; Khan et al., 1997; Twiner et al., 2001; Keppler et al., 2005). High levels of physiological variability have been reported among strains of *H. akashiwo*; with strains differing in toxicity (Tomas, 1978; Harrell, 1990), salinity and temperature tolerance (Smayda, 1998; Martinez et al., 2010; Strom et al., 2013), nutrient utilization (Fredrickson et al., 2011), viral susceptibility (Tarutani et al., 2000), cyst production (Han et al., 2002), and swimming behaviors (Bearon et al., 2004; Strom et al., 2013; Tobin et al., 2013). Given the observed physiological variation, it has been hypothesized that *H. akashiwo* is comprised of many ecotypes (Smayda, 1998). Moreover, salinity has been suggested as an influential factor in promoting *H. akashiwo* blooms (Tomas, 1978; Zhang et al., 2006; Fredrickson et al., 2011; Strom et al., 2013); therefore, we wanted to quantify the strain-specific behavioral response to both high and low salinity conditions. High genetic and inherent behavioral diversity was observed among all strains of *H. akashiwo*, in both low and high salinity. The diversity of swimming behaviors expressed resulted in unique, strain-specific vertical distributions of *H. akashiwo* in the water column. Further, these swimming behaviors were found to be a persistent trait, and were observed over both short and longer temporal scales. These data provide further documentation of the persistent intra-specific variability in phytoplankton traits that need to be considered in order adequately characterize the ecosystem function of genetically diverse phytoplankton populations.

## Materials and Methods

### Culture of *H. akashiwo* Strains

Seven strains of *H. akashiwo* were used in these experiments; five strains isolated from the west coast of the United States and two strains from the east coast (**Table 1**). Six strains originated from the National Center for Marine Algae and Microbiota. One strain was provided by Dr. S. Strom at the Shannon Point Marine Center (SPMC 135). Hereafter, we will refer to strains by their strain number. All strains were isolated within 4 years of one another, with the exception of 452, which was isolated in 1952. Strains 2808 and 2809 were isolated from the same water sample. Prior to conducting this study, single cells from all strains were re-isolated to ensure monocultures were used for both behavioral and genetic analyses.

**Table 1:** Strain name, isolation location and date, and carbon content (± standard deviation) for each *Heterosigma akashiwo* strain examined.

Strain name	Ocean Basin	Location	Date	Carbon content pg C cell^-1^ (SD)
CCMP2808	NE Pacific	Guemes Channel, WA, USA	2006	160 (3.7)^a^
CCMP2809	NE Pacific	Guemes Channel, WA, USA	2006	143 (3.4)^b^
CCMP3149	NE Pacific	Lummi Bay, WA, USA	2007	115 (3.1)
SPMC135	NE Pacific	Burrow’s Bay, WA, USA	2008	131 (3.1)^b^
CCMP3107^1^	NE Pacific	Nowish Inlet, BC, Canada	2008	162 (2.9)^a^
CCMP452	NW Atlantic	Long Island Sound, CT, USA	1952	155 (3.3)^ab^
CCMP3374^1^	NW Atlantic	Greenwich Cove, RI, USA	2010	97 (2.6)

*Strains labeled CCMP are from the National Center for Marine Algae and Microbiota, the strain labeled SPMC was provided by Dr. S. Strom at Shannon Point Marine Center, Western Washington University. ^1^Denote strains that were isolated by the authors. ^a,b,ab^Denote strains that do not have significantly different mean carbon content.*

Cultures were grown in 0.2 μm sterile-filtered autoclaved seawater enriched with f/2 nutrients minus silicate (f/2; Guillard, 1975). Cultures were maintained under a 12:12 h light:dark cycle at 15°C, salinity of ∼30 psu, and a light intensity of 80–100 μmol photon m^-2^ s^-1^. Cultures were transferred every 7–10 days to maintain exponential growth, and were not axenic. All experiments were conducted with exponentially growing cells. Cell counts were performed regularly to ensure exponential growth was maintained. Unless otherwise noted, cell concentrations were determined by microscope counts using samples fixed in 1% acid Lugol’s solution. For genetic analyses, genomic DNA was extracted from exponentially growing cells filtered onto a 47 mm, 0.2 μm filter (EMD Millipore, Billerica, MA, USA) using the DNeasy Plant Mini Kit (Qiagen, Valencia, CA, USA).

### 18S Sequencing

To confirm that all strains were identified correctly as *H. akashiwo*, the small subunit (18S) region of the ribosomal DNA was sequenced from each strain. Each 10 μl reaction mixture contained 5 μl BIO-X-ACT Short Mix (Bioline), 1 μl genomic DNA (∼3–5 ng), and 0.5 μmol L^-1^ each 18SA and 18SB universal primers (Medlin et al., 1988). Thermocycling consisted of a 3 min denaturation step at 94°C, 35 cycles of 94°C for 20 s, 60°C for 1 min, and 72°C for 2 min, followed by one cycle at 72°C for 10 min. Amplicons were sequenced in both directions on an ABI 3130xl using the DS-33 Dye Set (Applied Biosystems). Sequences were compared using CLC Workbench and Sequencher software programs. All sequences are available in GenBank (accession numbers: KT163009–KT163015).

### Microsatellite Genotyping

Six microsatellite loci (**Table 2**) were chosen for amplification following Nagai et al. (2006). Each microsatellite locus was PCR amplified from each *H. akashiwo* strain. PCR amplifications were performed in 10 μl volumes containing 3–5 ng of genomic DNA, 1x reaction buffer (Bioline), 0.1 mmol L^-1^ dNTPs (Bioline), 0.1 U Mango Taq DNA polymerase (Bioline), and 0.5 μmol L^-1^ each of forward and reverse primers with one primer labeled with either NED, HEX, or 6-FAM. The PCR cycling conditions were identical to those described in Nagai et al. (2006), briefly: 10 min at 94°C followed by 38 cycles of 30 s at 94°C, 30 s at 60°C, and 1 min at 72°C, and a final cycle of 5 min at 72°C. Amplification products were visualized on an ABI 3130xl Genetic Analyzer (Applied Biosystems) and were scored and sized using GeneMapper 4.0 software (Applied Biosystems) after capillary electrophoresis. The software program GENALEX 6.0 (Peakall and Smouse, 2006) was used for all genetic analyses, including observed and expected heterozygosity, allele frequency, departures from Hardy–Weinberg equilibrium, genetic distance, and relatedness. For all statistical comparisons, alpha was set to 0.05. Genetic distance between strains was calculated following Smouse and Peakall (1999). A principal coordinate analysis (PCoA) was used to visualize the genetic distance matrix between strains. Pairwise genetic relatedness between strains was determined in GENALEX following Lynch and Ritland (1999) and the output was used in a Mantel test (Mantel, 1967) to examine isolation by distance. Geographic distance between sampling sites was determined using the shortest ocean distance as calculated in Google Earth (Google Earth 7.1.2.2041). Given the wide range in geographic distances sampled, and the need to show the data on a log scale, the two samples collected from the same water sample were set to a geographic distance of 1 km.

**Table 2:** *Heterosigma akashiwo* microsatellite characteristics including microsatellite locus name (as in Nagai et al., 2006), number of genotypes, number of alleles, base pair size range, observed heterozygosity (*H_o_*), and expected heterozygosity (*H_e_*).

	Locus	# Genotypes	# Alleles	Size range (bp)	*H_o_*	*H_e_*
1	HaK30RR	5	4	238–249	0.571	0.704
2	HaK2R	4	4	213–221	0.286	0.459
3	HaK3R	6	7	183–215	0.429	0.816
4	HaK031RR	5	4	245–253	0.667	0.597
5	HaK37R	6	7	197–211	0.714	0.837
6	HaK17R	1	1	137	–	–
	Overall		4.5		0.533	0.683

*All microsatellites listed were examined in all seven *H. akashiwo* strains.*

### Calculation of Carbon Content

Strain specific carbon content was calculated from cell volume, assuming the shape of a prolate spheroid (Menden-Deuer and Lessard, 2000). For each strain, the length and width of 100 live cells were measured using a Nikon Eclipse E800 microscope equipped with image capture (Coriander) and image analysis software (ImageJ). Cells were measured live to avoid changes in cell size due to preservation (Menden-Deuer et al., 2001). Differences in carbon content among strains were compared using a one-way ANOVA.

### Behavioral Experiments

Behavioral experiments were conducted similar to those reported in Harvey and Menden-Deuer (2011, 2012). Briefly, to quantify population distributions and movement behaviors, a 30 cm tall, 5.5 cm wide, 800 mL octagonal, acrylic observational chamber was used. For all experiments, a linear salinity gradient from 0 to 30 psu was created in the chamber. Salinity decreased linearly from the bottom to the top of the tank, creating a stable water column not subject to convection. The same source water was used in all experiments and cultures. Each strain was filmed in triplicate.

Using a syringe, organisms were introduced at the bottom of the tank (at the equivalent salinity of 30 ppt) through silicone tubing with an internal diameter of 1 mm. Cells were introduced slowly at a rate of 10 mL min^-1^ to reduce stress to cells as well as disturbance to the water column. *H. akashiwo* was added to the tank for an average final concentration of 180 cells mL^-1^, and cells were allowed to acclimate for 10 min before filming started.

Two infrared sensitive cameras (Pixelink) with Nikon 60-mm Micro Nikkor lenses monitored a two-dimensional (2D) field of view approximately 1 cm × 1 cm × 1.5 cm. The cameras were mounted at a 45° angle with maximally overlapping fields of view to enable reconstruction of three-dimensional (3D) movement behaviors. All filming was conducted in the dark, to eliminate the potential for light-mediated behavioral responses. In order to image organisms, the chamber was illuminated with infrared (960 nm) light-emitting diodes (LEDs). Filming occurred at four evenly distributed vertical horizons, ∼5 cm apart. Swimming data from the lower and upper horizons were combined for analysis of high and low salinity swimming behaviors respectively. Each horizon was filmed for 1 min every hour for a 12 h period, and video was captured at 15 frames s^-1^ for a total of 1800 frames per observation. The order of filming the four horizons was initially randomized and then the same randomized order was used for all treatments, replicates, and time points.

To determine the vertical distribution and swimming behaviors of each strain of *H. akashiwo*, all videos were analyzed using the same protocol. The two-dimensional (2D) position of each organism in each frame of the stereo videos was determined, using automated ImageJ image-processing software to remove stationary background objects. The threshold was determined manually, so that background subtraction could be automated. Three-dimensional swimming paths were determined by first assembling 2D trajectories from Cartesian coordinates of each organism in each stereo frame and then joining 2D tracks based on matching space-time occurrence in the two 2D segments. Three-dimensional paths are necessary to avoid introducing biases in speed and direction estimates. Trajectories from all treatments were determined using the same video analysis and trajectory assembly parameters; more details are reported in Menden-Deuer and Grünbaum (2006).

Cell abundances in video images were determined by averaging the number of tracks per frame over the duration of the video yielding an average number of cells per frame and variation estimated across the 1800 frames in each 1-min segment. Swimming behaviors, including the x, y, and z velocity vectors and turning rates were calculated from 3D paths, subsampled at 0.10 s track intervals. Only trajectories that were longer than 3 s were used in the analysis. The upper and lower 1% tails of the frequency distributions of the swimming data were discarded before analysis to eliminate extreme outliers. The swimming behaviors and population abundances reported here are averages from the entire 12-h filming period averaged over the least (12 psu) or most (30 psu) saline portion of the tank respectively, unless otherwise stated. The Kolmogorov–Smirnov test (K–S test) was used to determine significant differences among abundance data, as well as among distributions of swimming behaviors. A K–S test was conducted contrasting each swimming metric measured separately (speed, turning rate, and vertical velocity) among all strains. In order to test whether the variability in movement behaviors among *H. akashiwo* was a result of intrinsic variability or reflective of the sampling size, the coefficient of variation (CV) for swimming behaviors was calculated iteratively, increasing the number of track intervals included in each iteration. Calculations of the cumulative CV for each strain were conducted on individual track intervals from all tracks observed in all replicates, horizons, and time points. From this pool, track intervals were chosen at random for the analysis. The abundance data are displayed graphically as the percent of the population at each horizon, calculated separately for each strain to allow for comparison between treatments. All statistical analyses were done on the absolute abundance data, not the percentages. For all analyses, the significance level was *p* < 0.05.

### Comparison between Genetic and Physiological Data

In order to test whether the genetic and physiological data collected were correlated, a Mantel test (Mantel, 1967) using a Pearson product-momentum correlation was performed on pair-wise dissimilarity matrices of the physiological parameter of swimming speed and the genetic relatedness matrix described above. The dissimilarity matrix of swimming speed was created by taking the absolute differences between mean swimming speeds of two strains. For each comparison, 999 permutations were run in R (using the vegan package), and the average Mantel statistic and *p*-value was obtained. Regression analysis was used to quantify the relationship between genetic relatedness and geographic distance, as well as genetic relatedness and absolute difference of mean swimming speed. Model II regression (independent variable measured with error) was used for these comparisons.

## Results

### Verification of Species Identification

The 18S sequences of all seven strains were identical to each other (100% agreement) and to GenBank accessions of *H. akashiwo* 18S, indicating that all strains used here represented the same species, *H. akashiwo*.

### Genetic Diversity among *H. akashiwo* Strains

Locus HaK17R was monomorphic and was excluded from the analysis. Of the remaining five loci, HaK37R and HaK3R were the most variable with seven alleles and six genotypes (**Table 2**). There were no significant departures from Hardy–Weinberg equilibrium among loci (*p* > 0.05).

**FIGURE 1:**
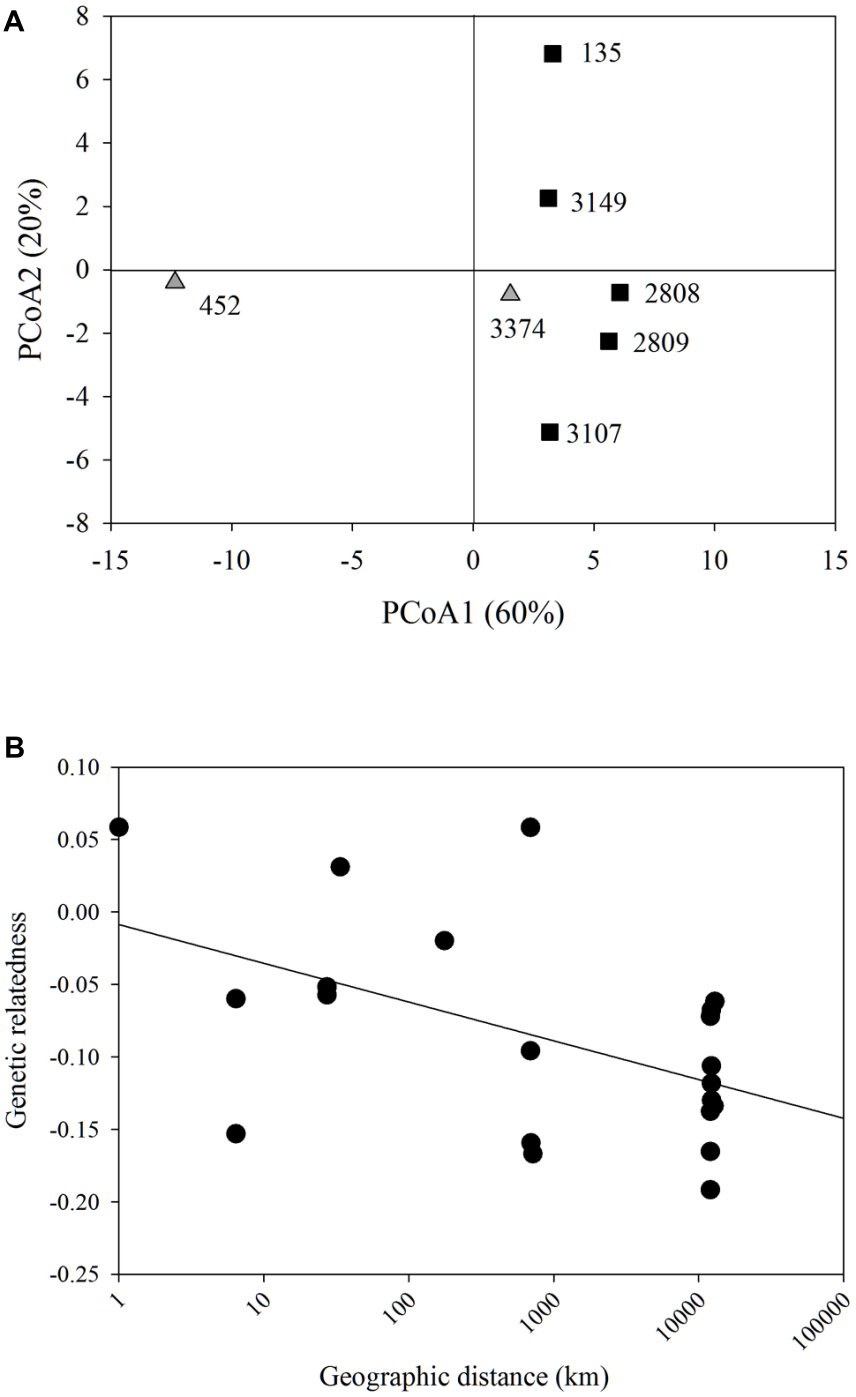
**Microsatellite analysis. (A)** Principal coordinate analysis of the genetic distance of all *Heterosigma akashiwo* strains examined. Strains do not cluster based on geographic origin (black squares – Pacific Northeast; gray triangles – Northwest Atlantic). PC1 and PC2 explained 80% of the variance in the data. **(B)** Model II linear regression for geographic distance (km) by genetic distance of all *H. akashiwo* strains (y = -0.0267x-0.0087, *r*^2^ = 0.26, *p* = 0.009).

Each strain had two alleles at one or more loci, indicating that these strains were diploid. Each strain had a unique multi-locus genotype, revealing that all seven strains represented different clonal lineages. PCoA of genetic distance revealed that the strain that had been in culture the longest, strain 452, was most genetically distant from all other strains along PC1 (**Figure 1A**). There was no clear clustering of strains isolated from the east or west coasts. PC1 and 2 explained 60 and 20%, respectively of the variation in the genetic distance matrix, with variation along PC1 largely due to differences among strain 452 and the remainder of the strains and PC2 capturing variation among the remaining strains. A comparison between genetic relatedness (Lynch and Ritland, 1999) and geographic distance revealed a significant negative slope. However, the relatively weak *r*^2^-value indicates that 3/4 of the variation observed is due to factors other than the relationship between genetic relatedness and geographic distance (*p* = 0.009; *r*^2^ = 0.26, **Figure 1B**).

### Swimming Behaviors and Distributions

**FIGURE 2:**
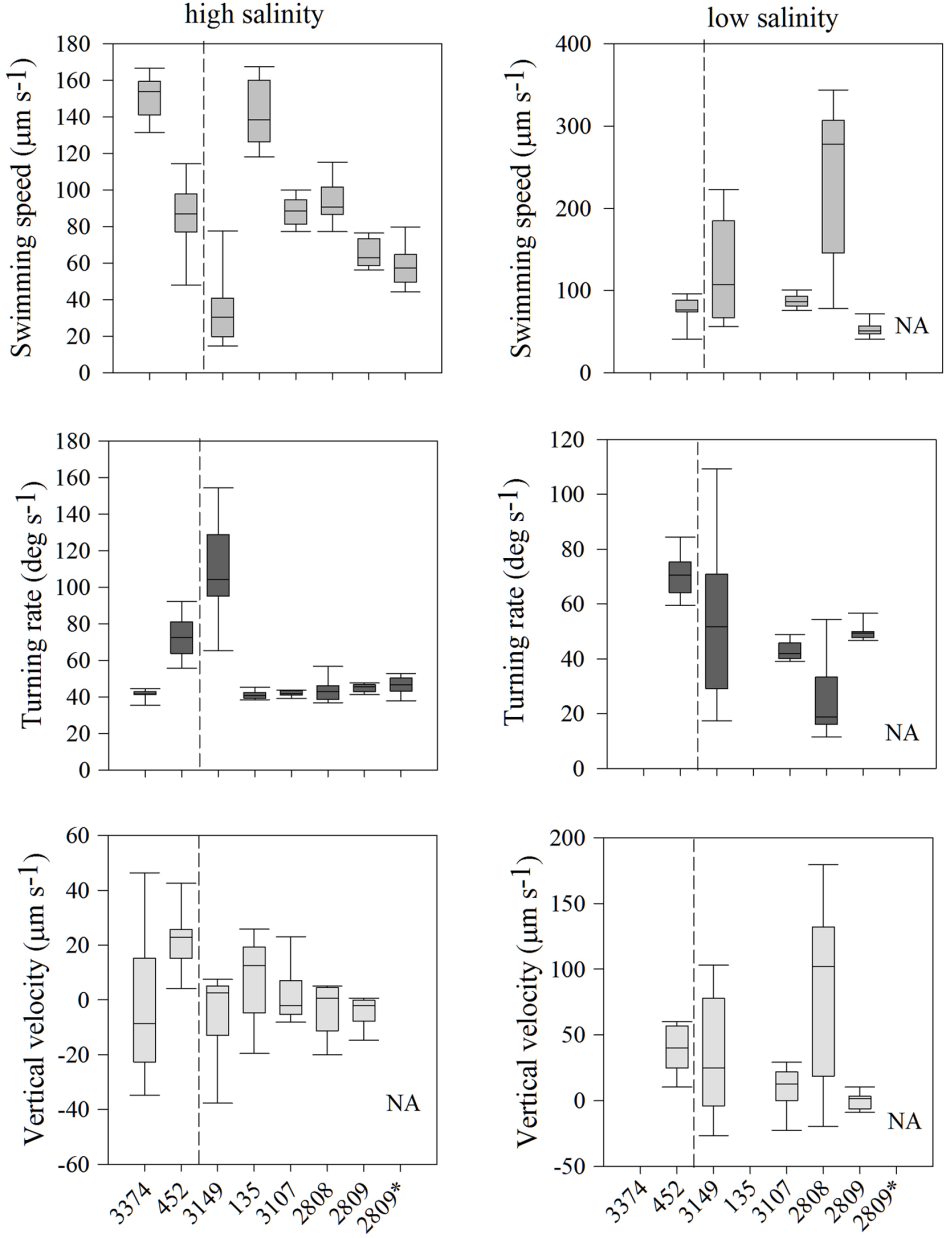
***Heterosigma akashiwo* movement statistics, including swimming speed (μm s^-1^; top row), turning rate (deg s^-1^; middle row), and vertical velocity (μm s^-1^; bottom row) in seven strains in high salinity (30 psu; left) and of five strains in low salinity (12 psu; right).** The asterisk next to strain 2809 indicates movement behavior data collected 2 years prior to the experiments conducted on all remaining strains. The dashed line separates strains isolated from the Northwest Atlantic (left of line) and from the Northeast Pacific (right of line). Error bars represent one standard deviation from the mean. NA indicates that strain 2809 was not measured under these conditions. Note difference in y-axis ranges.

Individuals within all seven strains exhibited significantly different distributions of swimming behaviors both among strains and with different salinities; all comparisons of swimming metrics between strains were significantly different (**Figure 2**; all *p* < 0.001, K–S test). In high salinity water (30 psu), there was a nearly five-fold difference in average swimming speeds among strains, from 33 ± 6 (strain 3149) to 151 ± 4 μm s^-1^ (strain 3374). Turning rates were less variable. Most strains had an average turning rate between 41 and 46 μm s^-1^ but strains 452 and 3149 had higher rates of 73 ± 4 and 110 ± 9 μm s^-1^, respectively. Vertical velocity was highly variable, with both upward and downward swimming observed, ranging from -2.8 ± 0.3 (net downward swimming) to 11 ± 0.3 μm s^-1^ (net upward swimming). Strain 452 swam fastest upward, and strain 3149 swam fastest downward.

In the behavioral response to low salinity water (12 psu), there were marked differences between strains relative to swimming in high salinity waters (**Figure 2**). Two strains (135 and 3374) avoided low salinity waters and, thus, cell concentrations were too low for behavioral analysis. For the remaining five strains, swimming speed in low salinity ranged from 85 ± 3 to 238 ± 10 μm s^-1^, turning rate from 24 ± 5 to 70 ± 2° s^-1^, and vertical velocity from 0.06 ± 0.1 to 81 ± 1.4 μm s^-1^. For strain 2808, swimming speed increased ∼150% and turning rate decreased 43% in low salinity water. In contrast, strain 3107 had virtually unchanged swimming behaviors irrespective of salinity. In low salinity water, strain 3149 exhibited swimming speeds and turning rates that were more similar to the overall mean swimming behaviors expressed by all strains in high salinity. Interestingly, for all strains, cells found in low salinity water displayed upward vertical velocities almost an order of magnitude larger than those observed in higher salinity. The exception was strain 2809, which displayed essentially no net vertical movement (0.06 ± 0.1 μm s^-1^).

**FIGURE 3:**
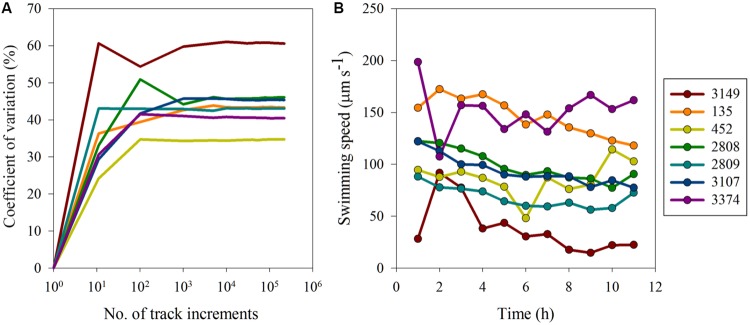
**Variability in *H. akashiwo* movement behaviors: **(A)** coefficient of variation (%) versus number of track increments and **(B)** average swimming speed (μm s^-1^) over time.** Error bars (calculated as one standard error from the mean) were very small and are obscured by the symbols.

Variability in movement behaviors were investigated on two scales (1) variability along the movement track of an individual and (2) variability in population-level mean movement behaviors over the experimental time period. With respect to individual level variability, the observed differences in swimming behavior were the result of inherent biological differences as opposed to methodological biases. For all strains a sample size of ∼1000 track intervals was sufficient to estimate the inherent variation in the data (**Figure 3A**) and inclusion of data from additional track intervals did not change the CV, indicating that a sub-sample of 1000 track intervals was sufficient to estimate the mean and associated inherent, sample size independent variation. Strain 3149 had the highest inherent variability, with a CV for swimming speed of almost 60%, whereas strain 452 had the lowest CV at ∼35%. Other strains had CVs that ranged between 37 and 50%. The CV as a function of sample size for other movement behaviors showed similar trends (data not shown). Overall, turning rate and vertical velocity variability was higher than inherent variability in swimming speed, with CVs ranging between 74 and 103% for turning rate and CV > 100% for all strains for vertical velocity. This indicates that while swimming speed remained consistent throughout the experiment, turning rate and vertical movement were more variable over time. However, with all movement metrics examined, a plateau in CV was reached after ∼1000 track intervals for all strains, showing that the observed variability was inherent to the strains’ motility pattern and independent of sample size.

Surprisingly, movement behaviors within strains were largely consistent both over hourly and yearly time scales. On an hourly time scale, strains exhibited similar movement behaviors over the course of the experimental period (**Figure 3B**). The CV among the means of swimming behaviors for each of the strains at each hourly time point was calculated to determine how well the data reflected the true mean of the swimming metrics. For swimming speed, the CV ranged from 12 to 19% in the majority of strains. However, strain 3149 exhibited more variable swimming over time, with a CV of 64%. On a yearly scale, the swimming speed and turning rate of strain 2809 had been measured under identical conditions 2 years prior to these experiments (see **Figure 2**). The CV in turning rate and swimming speed between the two measurements of strain 2809 are 2 and 13%, respectively. This is smaller than the variability observed on an hourly time scale for this strain (15%) and indicates high consistency of movement behaviors over time.

**FIGURE 4:**
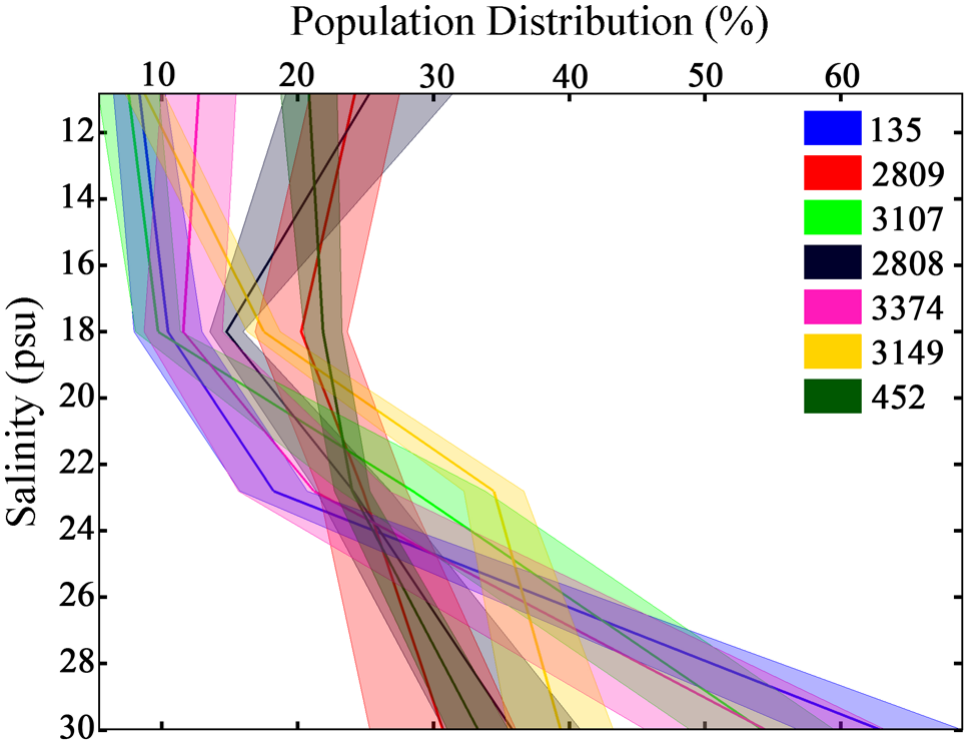
**Population distributions of *H. akashiwo* in the experimental tank over 12 h.** Height in the tank is expressed as salinity. Each color represents a different *H. akashiwo* strain as identified in the legend. The solid dark line represents the mean population distribution, and the width of each line represents one standard error of the mean; data was pooled from all time points and all three replicates. The high level of behavioral variability resulted in population-level variability in vertical distributions among strains.

As a consequence of the variance in swimming behaviors among strains, unique distributions of cells throughout the experimental tank were observed (**Figure 4**, all *p* < 0.001, K–S test). All strains had a higher mean abundance at the bottom, high salinity portion of the tank relative to the top, low salinity portion. The abundance of the population ranged from 30 ± 5 to 63 ± 6% at the bottom of the tank and from 7 ± 2 to 25 ± 6% at the top of the tank. No two strains had identical vertical distributions. The CV in cell abundance among horizons ranged from 17% in strain 2809 that was relatively homogeneously distributed vertically to 102% in strain 135 that had a population skewed toward the bottom of the tank.

### Carbon Content and Swimming Behaviors

Cell length ranged from 10 to 25 μm, with a CV of 9%. Average carbon content of the strains ranged from 97 ± 2.6 to 162 ± 2.9 pgC cell^-1^ (**Table 1**). The CV of carbon content among strains was 60%, whereas within strain CV was only 1–2%. Strains 3374 (97 ± 2.6 pgC cell^-1^) and 3149 (115 ± 3.1 pgC cell^-1^) had significantly lower carbon biomass than the other strains examined (*p* < 0.05 for all comparisons, ANOVA), and the remainder of the strains were not significantly different from at least one other strain.

**FIGURE 5:**
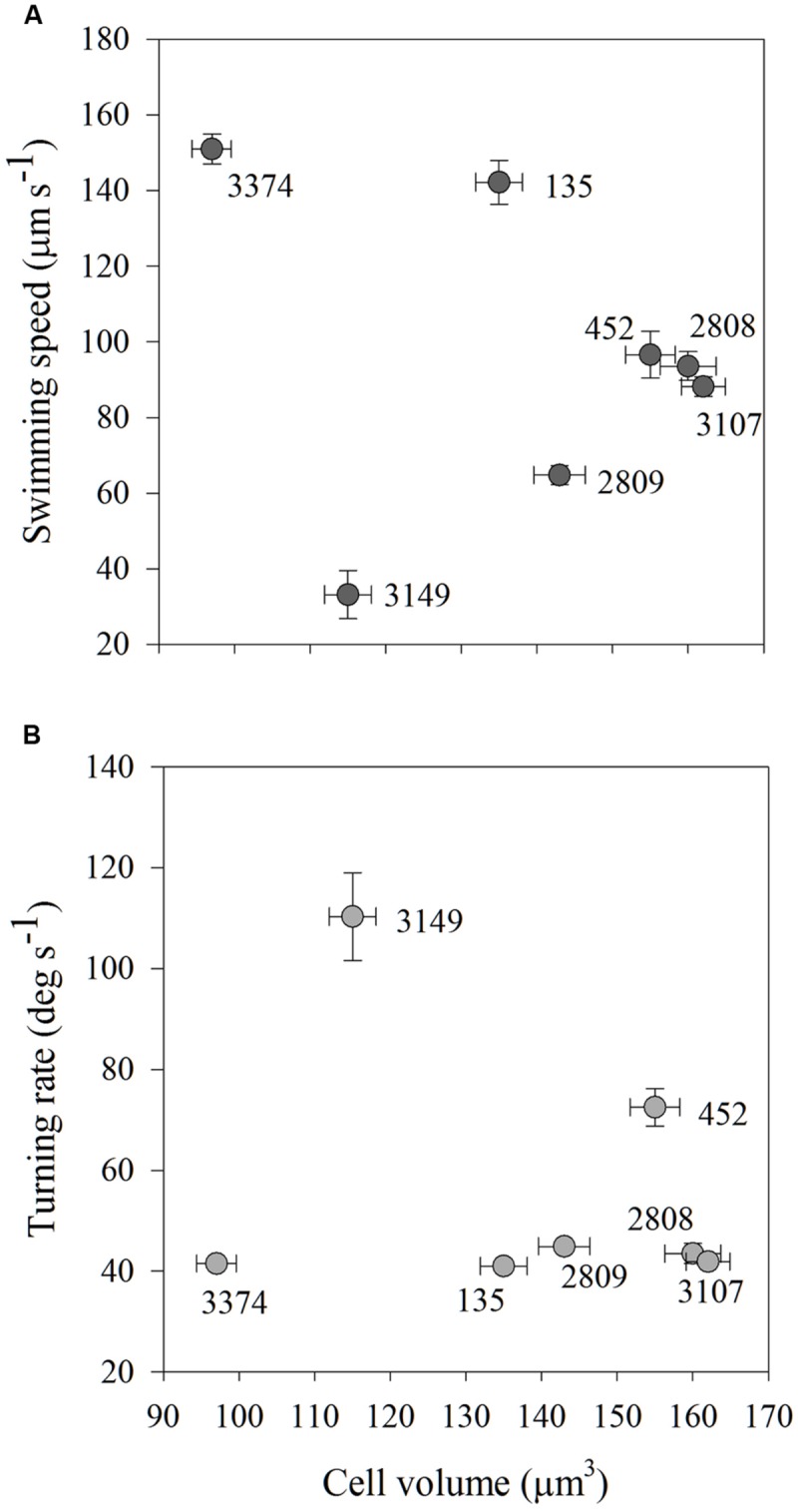
**Average swimming speed (μm s^-1^; **A**) and turning rate (deg s^-1^; **B**) in high salinity water of each strain of *H. akashiwo* in relation to its cell volume (μm^3^).** There were no associations between cell size and cell motility indicating that within-strain differences in motility were driven by mechanisms other than cell size. Error bars are one standard deviation from the mean.

As carbon content is a function of cell biovolume, average carbon content and average swimming speed and turning rate were compared to determine if cell size could explain the variability in movement behaviors among the strains. There were no significant associations among average carbon content and average swimming speed or turning rate for any of the strains examined (**Figures 5A,B**).

### Correlations between Genetic and Physiological Data

**FIGURE 6:**
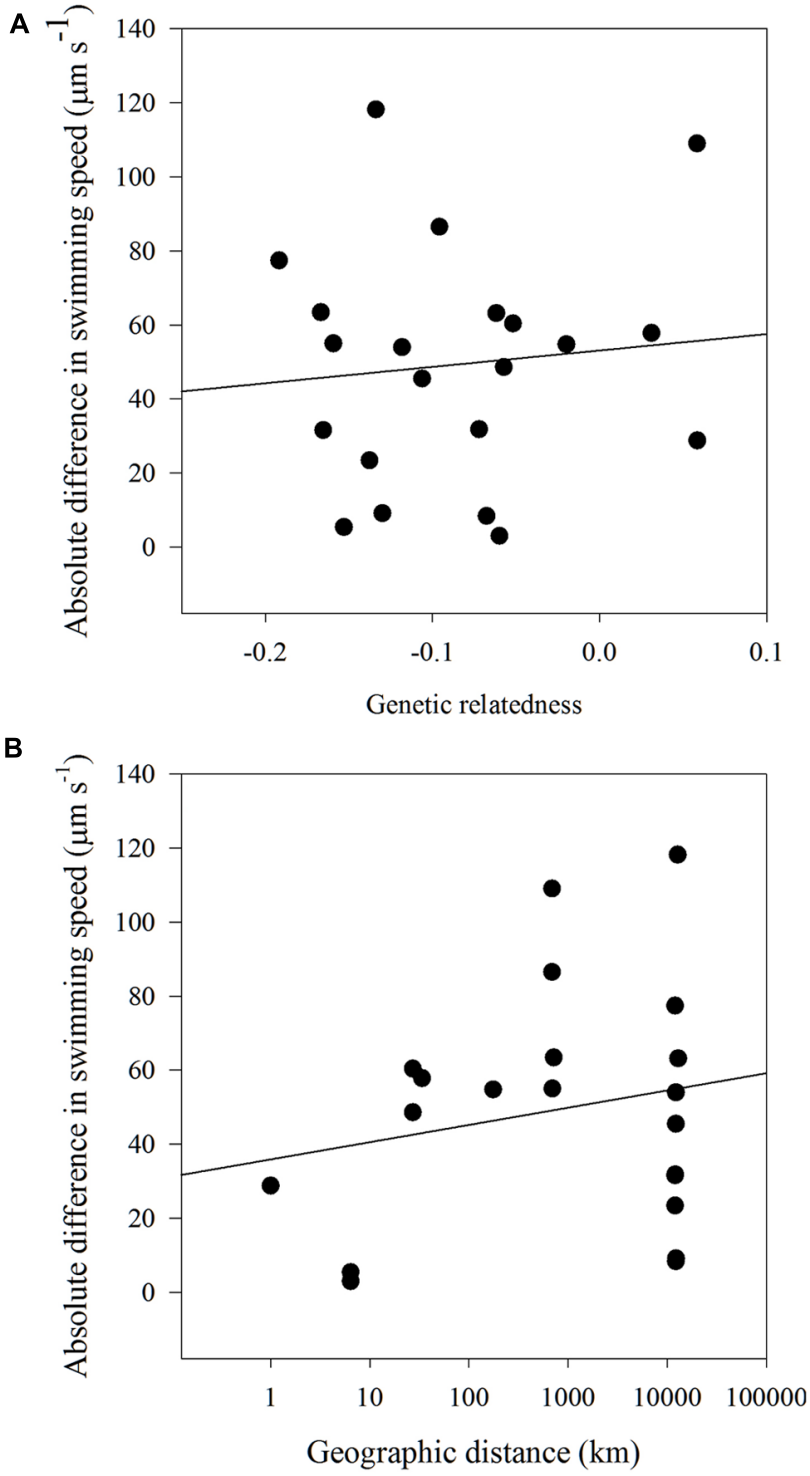
**Model II linear regression for **(A)** absolute difference in swimming speed (μm s^-1^) and genetic relatedness (y = 44.2x+53.1, *r*^2^ = 0.01, *p* = 0.66) and **(B)** absolute difference in swimming speed (μm s^-1^) and geographic distance (km) for all *H. akashiwo* strains (y = 4.6x+35.88, *r*^2^ = 0.04, *p* = 0.38)**.

There were no significant correlations found between the dissimilarity matrices of swimming speed and genetic relatedness (Mantel statistic = 0.028; *p* = 0.45). No significant correlations were found between the dissimilarity matrices of swimming speed and geographic distance (Mantel statistic = -0.028; *p* = 0.43) and a model II regression analysis between the absolute difference in swimming speed and either genetic relatedness or geographic distance revealed no relationship between the two factors (**Figures 6A,B**).

## Discussion

Trait-based approaches have been utilized increasingly in phytoplankton ecology to, amongst others, predict phytoplankton community organization (Litchman and Klausmeier, 2008). In this study, the trait of motility was examined, revealing significant behavioral differences among genetically distinct strains of a single species, *H. akashiwo*. These results demonstrate that one strain cannot be considered representative of a species, and that each of these strains will have different distributions in the water column and thus, different encounter rates with resources and predators. Our results make clear the importance of not only investigating multiple isolates of a species to characterize the range of a particular phenotypic trait, but they also highlight the potential of motility as a persistent integral functional trait that can deliver high resolution distinction where broad characteristics, such as cell size, fail to deliver appropriate distinction.

### Genetic Variability

The six microsatellites adapted from Nagai et al. (2006) and used in these experiments had variable levels of polymorphism. One locus, HaK17R, was monomorphic and thus not informative in distinguishing among strains. In contrast, Nagai et al. (2006) identified six alleles at this locus using strains isolated from Hiroshima Bay, Japan. The differences between the two studies may be due to the fact that isolates from different geographic regions were examined. All other loci examined had levels of polymorphism similar to those identified previously and none of the loci deviated from Hardy–Weinberg equilibrium. In contrast, locus HaK37R had previously been identified as having significant heterozygote deficiency, again using isolates collected from a different geographic region than those examined here (Nagai et al., 2006).

Each *H. akashiwo* strain examined here had a unique multi-locus genotype and thus all strains were genetically distinct from one another. These findings validate the usefulness of these loci (Nagai et al., 2006) for distinguishing among clonal lineages isolated from field samples. The high degree of clonal diversity observed in this raphidophyte is consistent with previous literature showing high degrees of clonal diversity in a range of phytoplankton taxa including dinoflagellates, diatoms, and coccolithophores (e.g., Rynearson and Armbrust, 2005; Iglesias-Rodriguez et al., 2006; Frommlet and Iglesias-Rodriguez, 2008; Richlen et al., 2012). The microsatellite data revealed that pairwise genetic distance varied among strains, but strains were not equally differentiated from each other. For example, strains 2808 and 2809, isolated from the same water sample, were least differentiated. Conversely, genetic distance was greatest between strain 452 and the other strains examined, including strain 3374, which was also sampled from the NW Atlantic. The genetic distance between 452 and all other strains may be related to the length of time 452 has been in culture (over 50 years) versus the remainder of the strains, which were all isolated within the last 4 years.

Remarkably, geographic distance was not a good predictor of genetic relatedness among strains. For example, strains that were collected on the order of 10 km apart (2808 and 135; 2808, 2809, and 3149) were as closely related as those collected 10,000 km apart (3374 and 135; 452 and 3149). Although the regression of genetic relatedness and geographic distance yielded a significant negative slope, the small *r*^2^-value (0.26) indicated a high level of variability in the relationship between genetic relatedness and geographic distance and, likely, factors other than distance played an important role. This result contrasts with a previous study examining genetic isolation over basin-scale distances in the phytoplankton, where the diatom *Pseudo-nitzschia pungens* displayed strong isolation by distance with a significant negative slope and an *r*^2^-value of 0.76 (Casteleyn et al., 2010). It was hypothesized that the pattern of isolation by distance in *P. pungens* revealed limits to gene flow in the phytoplankton, despite their potential for nearly unlimited dispersal with tides and currents. The small data set analyzed here indicates that limited gene flow may not be the norm amongst all phytoplankton. Differences in genetic connectivity among phytoplankton may be due to variations in their ability to traverse “poor” habitats, which would likely be encountered during long-distance dispersal. Additional factors that play a role in shaping the genetic connectivity of populations include rates of sexual reproduction, population size, and strength of selection (Hartl and Clark, 2007). Indeed, very little is known about these factors in either diatoms or raphidophytes, outside of the fact that both are able to reproduce sexually (Round et al., 1990; Imai, 2003).

Behavioral and genetic variation did not correlate among *H. akashiwo* strains; there was no association between the clustering of strains by movement traits and genetic relatedness. Lack of a correlation between defined genotypes and phenotypic diversity has been previously observed, in studies investigating the genetic and toxicity variability in the dinoflagellate, *Alexandrium tamarense* (Alpermann et al., 2010). A lack of a correlation implies that the traits can be useful to characterize phenotypic variability in this species but are not linked such that one trait could be predicted from the other. As microsatellites and behavior do not correlate in our experiments, they both could be used as traits to identify specific strains, and provide a much finer resolution compared to cell size, which would not have allowed us to distinguish amongst the strains examined here.

### Inherent Behavioral Variability

All strains of *H. akashiwo* observed exhibited significantly different movement behaviors relative to one another. Behavioral variability among strains of *H. akashiwo* has been observed previously, and swimming speeds have been reported to range across an order of magnitude (20–150 μm s^-1^; Throndsen, 1973; Bauerfeind et al., 1986; Bearon et al., 2004; Tobin et al., 2013). The movement behaviors observed in this study similarly span an order of magnitude. In addition, we observed, for most strains, an increase in swimming speed when in low salinity water (12 psu) relative to high salinity water (30 psu). Prior work has shown that both absolute salinity and acclimation to it influence swimming behavior (Strom et al., 2013), as does presence of predators (Harvey and Menden-Deuer, 2012). Thus, while movement behaviors are variable they are consistent and distinguish strains.

Above a minimum sample size of ∼1000 tracks, a constant CV as a function of sample size was observed among all strains examined, this indicates that the underlying variability in movement behaviors measured in this study was driven by biological characteristics, rather than differences due to sample size. Having eliminated sample size as a possible contributor to the degree of variability in movements observed, our results show that the different strains harbored different degrees of inherent variability with respect to their movement behaviors. Further, movement behaviors of *H. akashiwo* appeared to have considerable consistency, with identical values observed from hour to hour and even across years; these observations agree well with prior studies on the same strain (2809; Kim et al., 2013). Moreover, the movements of strain 452 made here bear a striking similarity to those published previously and made independently of our own measurements. Experiments that have used strain 452 previously have reported that mean vertical velocity of this strain was 36 μm s^-1^ (Tobin et al., 2013) and ranged from 35 to 60 μm s^-1^ (Bearon et al., 2004). Similarly, the average vertical velocity of this strain (averaged across all data points) in our experiments was 36 μm s^-1^ and ranged from 25 to 72 μm s^-1^. This agreement of swimming metrics measured from studies conducted in different laboratories over several years with different subcultures of the same strain is less than the variation observed among strains within the same study, both here and elsewhere (e.g., Bearon et al., 2004; Tobin et al., 2013). Comparisons of maximum gross speed of strain 452 among the studies was more variable, however, speeds measured in the current experiment are still within the range of speeds measured previously for this strain (Bearon et al., 2004; Tobin et al., 2013). The repeatability of estimating movement behaviors consistently over time and culture conditions is surprising, given that cultivation of phytoplankton in the laboratory can significantly change the initial phenotype of the cell isolated, and runs the risk of adaptation to culturing conditions (Lakeman et al., 2009; Berge et al., 2012). Yet, the observed consensus of similar movement behaviors within the same strain over space and time, suggests that *H. akashiwo* behavioral differences are inherent and based on intrinsic genetic or physiological differences that are insensitive to peculiarities of the specific, though similar, culturing conditions and methods utilized by different researchers. This consistency suggests movement behaviors are a robust trait that reliably identifies a strain.

Strain-specific behaviors resulted in strain-specific, vertically structured population distributions in the water column in relation to a salinity gradient. Strain-specific differences in population distributions based on movement behavior differences have been hypothesized and modeled (Bearon et al., 2004). The present study validates those hypotheses and demonstrates the direct link between individual-level motility behaviors to genetically distinct population distributions. Theoretically- derived hypotheses suggest that no single movement behavior is best suited to exploit all potential resource conditions (Grünbaum, 1998). Additionally, intra-specific variability is hypothesized to support the coexistence of many identical but inherently variable species or strains (Menden-Deuer and Rowlett, 2014). Thus, a wide array of behavioral phenotypes would increase *H. akashiwo’s* niche and benefit the species, by allowing *H. akashiwo* to out compete other co-occurring phytoplankton species that may not share the same level of intra-specific variability and capacity to responsed to fluctuating resources. A behaviorally diverse population would be able to survive over a broader range of environmental conditions than a behaviorally invariant one. Similar observations of inherent variability have been made for the dinoflagellate *Akashiwo sanguinea* and been suggested to promote the survival of this relatively slow growing species for many months along the entirety of the US west coast (Menden-Deuer and Montalbano, 2015).

### Cell Size Variability

Cell size is often suggested as a master trait to predict community composition along environmental gradients and predator consumption rates (Litchman et al., 2010). Previous studies have shown that maximal swimming velocities increase with size (Sommer, 1988). However, in our experiments there was no significant association among movement behaviors and strain biovolume, although size range is naturally small among strains of the same species relative to the order of magnitude differences in cell size often invoked in comparative studies. As with other raphidophytes, *H. akashiwo* has two flagella, and both can be used to provide the cell the ability to change the parameters of their helical swimming path (Fenchel, 2001). Therefore, the variability in movement behaviors among isolates could have resulted from differences in flagella morphology or the cell’s physiological capacity for propulsion (Raven and Richardson, 1984; Fenchel, 2001; Roberts and Deacon, 2002). The current findings indicate that cell size cannot be used to estimate movement behaviors nor to predict dispersal and distribution among strains of *H. akashiwo.* However, over order of magnitude size ranges, when moving from small to large organisms, Reynolds number increases do change the optimal shape for locomotion (e.g., spherical cells to more streamlined forms Litchman et al., 2013). Thus, while size may be an important metric in determining motility patterns over a large size range, it does not appear to dictate intra-specific patterns of variation, within the much smaller size range covered by strains of the same species. Motility metrics may be an additional trait, independent of cell size, which could be valuable for use in trait-based models that seek to characterize phytoplankton community dynamics.

## Conclusion

A struggle in the formulation of trait-based phytoplankton population models lies in the difficulty involved with identifying traits that adequately resolve variability among individuals, allowing the ecological niche of a particular species to be defined. Here we show that both genetic differentiation and motility are highly variable in *H. akashiwo.* Determination of phytoplankton motility provided the opportunity to characterize phenotypic variation among individuals, and demonstrate that this variation has ramifications at the population level, for example for the population distribution in the water column. While routine measurements of swimming behaviors using current methodology may be prohibitive, future developments in coupling *in situ* camera systems (e.g., Kane et al., in review) with ocean observing systems may afford the ability to make such measurements for the analysis of *in situ* plankton composition. These traits may be useful to characterize within-species variation where other metrics, such as cell size, fail to deliver the appropriate resolution. Ultimately, these traits may also prove useful in distinguishing among species. Thus, movement behaviors as well as genetic variability are high-resolution traits that can adequately capture intra-specific variability in order to better understand and predict the structure and function of plankton communities.

## Author Contributions

ELH, SM-D, and TAR designed the experiments, ELH collected the data, all authors analyzed and wrote the paper.

## Conflict of Interest Statement

The authors declare that the research was conducted in the absence of any commercial or financial relationships that could be construed as a potential conflict of interest.
